# A Qualitative Evaluation of Holiday Breakfast Clubs in the UK: Views of Adult Attendees, Children, and Staff

**DOI:** 10.3389/fpubh.2015.00199

**Published:** 2015-08-13

**Authors:** Margaret Anne Defeyter, Pamela Louise Graham, Kate Prince

**Affiliations:** ^1^Department of Psychology, Northumbria University, Newcastle upon Tyne, UK; ^2^Kellogg’s, Manchester, UK

**Keywords:** summer meals, breakfast, children, families, food poverty

## Abstract

Across the UK, 1.3 million children access free school meals for around 38 weeks of the year. However, during school holidays, many families face considerable difficulties in providing a consistent and nutritious supply of food for their children, particularly during the extended summer break. In an effort to address this issue, a number of community-based breakfast clubs were set-up across the North West of England and in Northern Ireland where people could access a free breakfast meal during the summer holidays. Qualitative interviews were carried out with 17 children, 18 adult attendees, and 15 breakfast club staff to determine the uses and impacts associated with holiday breakfast club participation and to investigate potential areas for future development of holiday food provision. Findings highlighted a need for holiday food provision and revealed a multitude of nutritional, social, and financial benefits for those who accessed holiday breakfast clubs. Areas for further development and investigation are discussed in addition to implications for UK food and educational policies.

## Introduction

Food poverty has been defined as “The inability to afford or to have access to food to make up a healthy diet” (1) (p. 7). While approximately 98% of those affected by such circumstances are situated in developing countries, such as Asia, Africa, and Latin America (2), the number of UK families having to rely on food aid has increased dramatically in recent years. Statistics provided by the UK’s main food aid providers, the Trussell Trust, Fareshare and Food Cycle, suggest that there was a 54% increase in the number of families accessing food banks from 2012/2013 to 2013/2014. This increased need for food aid has been attributed to a number of financial issues including increased fuel and food costs but delays and changes to state benefit allowances, which led to a dramatic reduction in income for many households, have been proposed as a key reason for increased food aid access (3). However, food poverty is not something that is unique to those families living on very low incomes (4); 62% of children in poverty live in households with at least one working parent (5). Moreover, food aid figures do not reflect the true extent of the problem as not every family experiencing food poverty will go on to access food aid (6).

Sufficient access to food is recognized as a basic human right. Article 25 of the Universal Declaration of Human Rights (7) states that “Everyone has the right to a standard of living adequate for the health and wellbeing of himself, and of his family”^1^, which includes adequate food. Additionally, the United Nations Committee on Economic, Social, and Cultural Rights (8) have since proposed that an adequate supply of food is critical to the fulfillment of other human rights and is intrinsic to human dignity.

The importance of nutritious food, particularly for young children, was recently acknowledged by the UK Government with the introduction of universal infant free school meals (9). Under the scheme, which was introduced in September 2014, all children in Key Stage 1 (aged 4–7 years) in primary schools across England are able to access school lunch at no cost to their parent or carer. Additionally, the UK Government also invested £3 million into the set-up of breakfast clubs in schools residing in low-income areas of England (i.e., those schools with a high proportion of pupils entitled to free school meals). The impact of these schemes is still under investigation but previous research into school food provision has shown that children who access school food increase their intake of healthy food items (10, 11) and key vitamins and minerals (12). However, under most school policies across the UK, school food is provided to children during term time only and no additional provision is in place during the school holidays. This causes considerable challenges for families, especially during the long summer break when they need to provide extra meals for their school-aged children for a duration of 6–7 weeks. In a report by Gill and Sharma (13), interviews with low-income parents revealed that the summer break is a time fraught with financial difficulties, typically owing to the burden of having to provide extra meals for their children. Parents discussed how they made efforts to stretch their food budgets by purchasing food that was cheap but generally unhealthy with cost having to take priority over nutritional adequacy. Moreover, the provision of additional food during the holidays was found to have wider reaching implications as some parents stated that they had missed household bill payments to be able to allocate money to the family food budget. Also, a lack of food meant that children were limited socially as they were unable to invite friends over to their houses to play as their parents were unable to extend their household food provision to feed additional children.

Given that children’s food intake is vital to their health status as well as their ongoing development (14), children’s dietary habits during the school holidays warrant attention. According to the Food Research and Action Centre (FRAC) (15) children’s meal intake during the summer break from school can be sporadic, often consisting of foods lower in nutritional value than they would receive at school during term time. FRAC pointed out that poor nutritional intake combined with more sedentary behavior throughout the summer leaves children exposed to the risk of weight gain and obesity. Research utilizing data from the Early Childhood Longitudinal Study-Kindergarten Cohort showed increased rates of growth in body mass index (BMI) over the summer compared to term time when gains in BMI slowed down. Moreover, changes in BMI were found to be less variable during term time than they were during the summer, suggesting that rapid changes in BMI are less likely to occur during the school term (16).

In an effort to address the inconsistencies in children’s dietary habits between term time and the summer break, the United States Department of Agriculture (USDA) fund summer meals programs allowing children to access meals and enrichment activities in safe, community-based environments during the extended summer break from school; though research investigating the effectiveness of such schemes is limited. Prior evaluations of summer feeding schemes have focused primarily on the operational aspects of programs; for example, as part of a summer meal survey carried out by Share Our Strength and APCO Insights (17), interviews with low-income families showed that most were interested in summer meals but some felt that they did not need them. The provision of healthy meals in a safe and trustworthy environment was a key consideration for parents while children valued fun activities and social opportunities.

Although summer feeding programs aim to “help kids who rely on free and reduced-priced school meals to continue receiving healthy meals during the summer”^2^, no published research, to date, has investigated the impact of this food provision on key developmental outcomes relating to children’s health and wellbeing. Moreover, unlike in the USA, the UK does not have an established summer feeding program in place and there are no formal records relating to the set-up and implementation of such clubs thus there are no academic studies on the provision of UK-based summer feeding programs investigating outcomes relating to participation. This is surprising given that recent census data from the Department for Education (18) showed that 15.2% of pupils in England access free school lunches during term time and, as mentioned previously, families of children accessing free school meals encounter considerable difficulties during the summer break (13).

In July 2014, six breakfast clubs were set-up in community venues across the North West of England and Northern Ireland. While breakfast clubs have been in place in UK schools since 1990s (10), the implementation of holiday breakfast clubs is relatively new. The new, pilot breakfast scheme under investigation was, to our knowledge, the first scheme of this kind to be implemented across multiple areas of the UK. The clubs provided a range of breakfast foods including cereals, toast, and fruit to children and adults free of charge. Some of the clubs also provided activities for attendees to participate in.

### Objectives

The current study set out to address the dearth in the research literature in the area of UK holiday food provision by evaluating these six holiday breakfast clubs. Utilizing a qualitative approach to draw on the views of children and adults, the current study set out to investigate the uses, impacts, and areas for future development of holiday breakfast clubs in the UK.

## Method

### Approach

The views of adults and children were obtained through face-to-face, semi-structured interviews. A semi-structured interview approach was adopted as previous research has shown this to be an adequate method of successfully obtaining information from adults and children on the topic of breakfast clubs (19, 20). Additionally, an interview approach is inclusive; it allows a diverse range of participants to give their views, regardless of age and educational ability.

### Participants

A purposive sampling method was utilized for the current investigation. Purposive sampling refers to the recruitment of participants according to a particular characteristic of interest (21). It has been found to be a robust method of sampling and is considered crucial to the quality of qualitative data collection (22).

Seventeen children, 18 adult participants, and 15 breakfast club staff were purposively sampled as they all attended or contributed towards the day-to-day running of a holiday breakfast club and would therefore be likely to have views on it. Participants were recruited from six holiday breakfast clubs; five of which were based in the North West of England and one that was based in Northern Ireland. The characteristics of each breakfast club are shown in Table 1. In order to uphold participant anonymity, clubs are referred to by number rather than the area in which they resided.

**Table 1:** **Details of holiday breakfast clubs**.

Club	Setting	Days and times of operation	Typical rates of attendance	Breakfast provided	Activities
1	Community building	Monday–Friday 9:00 a.m.–11:00 a.m.	40–50 attendees per day. Open to children and families. Welcomed children without parental supervision	Cereals, toast, juices, milk, fruit, hot drinks	No activities ran as part of the breakfast club but many children went from breakfast club to other activities provided in the community
2	Community building	Monday–Friday 9:30 a.m.–11:30 a.m.	50–70 attendees per day. Open to children and families. Mainly attended by young children with parents and child minders	Cereals, toast, juices, milk, fruit, hot drinks	Community building based within a park so there was space for indoor crafts and games as well as outdoor sports and play. Various community members ran activities
			Children had to be supervised by a parent or carer		
3	Community church building	Monday and Friday 9:15 a.m.–10:15 a.m.	Around 30 attendees. Parents with young children. Club ran at the time that an under 5’s group typically took place during term time. Children had to be accompanied by a parent or carer	Cereals, toast, juices, milk, fruit, hot drinks	Table top activities, such as drawing and construction
4	Food bank	Monday–Wednesday 9:00 a.m.–11:00 a.m.	Only 1 person attended the breakfast club on 1 day throughout its period of operation	Cereals, toast, beans, juices, milk	Planned to run craft activities
			Children had to be supervised by a parent or carer		
5	Food bank	Monday–Friday 9:30 a.m.–11:30 a.m.	Initially had 0 attendees but opened club up to the wider community (i.e., not just families with children) and attracted around 4–6 people from the community	Cereals, toast, juices, milk, fruit, hot drinks	Had drawing and games available plus a children’s DVD available
6	Started in Food Bank building but moved to larger church building after a couple of weeks to accommodate greater numbers	Monday, Wednesday, and Friday 10:00 a.m.–12:00 p.m.	Around 30 attendees	Cereals, toast, juices, milk, fruit, yogurts	Mainly craft activities. Breakfast club attracted help from external organizations to support activities and club staff took some children to activities outside of breakfast club to encourage activity engagement
			Referrals from various organizations including social services. Parents and children were invited to attend but parents could leave their children and return to collect them after the club, which most did		

Participating children were aged between 4 and 15 years (Mean age = 9 years); 11 were female and 6 were male. Four children were recruited from a church-based breakfast club (Club 3), seven were recruited from community-based breakfast clubs (5 from Club 1 and 2 from Club 2), and six children were recruited from a food bank club (Club 6). No children attended Club 4 and the individual child in attendance at Club 5 chose not be interviewed.

Breakfast clubs were initially aimed at children and families but it became apparent that some of the breakfast clubs were wider reaching and attracted adult participants that had no dependent children. The adult participant group consisted of 12 parents, 5 of whom were recruited from Club 1, 2 were recruited from Club 3, 1 from Club 5, 2 from Club 2, and 2 from Club 6. Additionally, three child minders (i.e., people who look after other people’s children in their own homes) were recruited from Club 2; one grandparent was recruited from Club 1; and two adults who had been referred to the club by external agencies were recruited from Club 5. No adult participants attended Club 4. Demographic details collected from the 18 adult participants are presented in Table 2.

**Table 2:** **Demographic information from adult participants**.

Demographic	Characteristic	Percentage of adults identifying with each characteristic (%)
Age group	18–25 years	5.6
	26–35 years	27.7
	36–45 years	38.9
	46–55 years	16.7
	55–70 years	11.1
Ethnicity	White British	83.3
	White Irish	11.1
	Did not disclose	5.6
Marital status	Single	44.4
	Living with partner	5.6
	Married	38.9
	Divorced	11.1
Employment status	Unemployed	44.4
	Employed part time	5.5
	Employed full time	22.3
	Self employed	16.7
	Too ill to work	11.1

Finally, 2 male and 13 female staff members were recruited to participate in the current study. Four members of staff were recruited from Club 4; two staff were recruited from Club 2; two from Club 3; three from Club 1, two from Club 5, and two from Club 6. Demographic information collected from staff members is outlined in Table 3.

**Table 3:** **Demographic information from staff**.

Demographic	Characteristic	Percentage of adults identifying with each characteristic (%)
Age group	18–25 years	66.6
	36–45 years	40
	46–55 years	20
	55–70 years	33.3
Employment status	Volunteer	13.3
	Employed part time	26.7
	Employed full time	26.7
	Self employed	13.3
	Retired	20

### Materials

Three separate interview schedules were designed to guide discussions with adults, children, and staff. Different interview schedules were put together to ensure that the wording used was appropriate for the group being interviewed, though the content of the questions remained consistent across all groups. The interview questions focused on What happens at breakfast clubs and why do people attend? What difference do breakfast clubs make to children, adults, and communities? Are there any issues associated with the utilization of breakfast clubs? What are the positive aspects of breakfast clubs? What could be done to improve breakfast clubs? How would circumstances have been different if breakfast clubs had not been available?

### Procedure

Following ethical approval from the University ethics committee, the person in charge of each participating breakfast club provided consent for all interviews to take place on their breakfast club premises. Opt-in consent was obtained for all adult participants and staff prior to commencement of interviews. Parental consent was obtained for all children prior to their participation and all children provided assent. All interviews took place in a quiet space on breakfast club premises at a suitable time to each individual and the requirements of the breakfast clubs. A semi-structured format was used throughout to allow the interviewer to gain further insight into points of interest and to seek clarification from participants when necessary. All interviews were audio recorded and transcribed verbatim for subsequent thematic analysis.

### Analysis

Each interview transcript was subject to thematic analysis, which is a qualitative method of analysis that provides “a rich and detailed, yet complex account of data” (23) (p. 5). The current analysis was not reliant on a theoretical framework and did not set out to develop a theoretical model but aimed to present a detailed account of holiday breakfast clubs according to the children and adults involved in them, thus thematic analysis was deemed the most appropriate method of analysis in this instance.

Each transcript was repeatedly read and key points pertaining to the research questions were highlighted. Highlighted quotes were labeled to indicate the topics they referred to then similar topics were grouped to form themes. Appropriate theme headings were generated to summarize the content of each theme. An inductive approach to thematic analysis was adopted as there is currently no existing framework on the uses, impacts, and developments of holiday breakfast clubs on which the analysis could have been based. A second coder analyzed around 10% of the data and a Kappa Coefficient was calculated in order to determine the level of agreement between two independent coders for a sample of data. This is a recognized method of determining reliability in qualitative investigations (24). Agreement between the first and second coder was found to be very good (Cohen’s κ = 0.792; *p* < 0.001).

## Findings

Holiday breakfast clubs were believed to offer a multitude of benefits to those who attended, with participants keen to see clubs rolled out on a wider scale throughout the year. Participants offered a unique insight into holiday breakfast clubs and provided views on areas that require further consideration in future development of holiday food provision. A detailed analysis of the themes identified by staff, adult attendees, and children is subsequently presented with example quotes to support each theme.

### What are the uses, impacts, and areas for future development of holiday breakfast clubs according to staff?

The views of breakfast club staff are important but underrepresented within the research literature (19). Given that staff are involved in the day-to-day running of the breakfast clubs and work directly with those accessing the clubs as well as external, supporting organizations, they are uniquely positioned to provide an accurate view of the uses and impacts of breakfast clubs and how best to develop such provision in future.

Six key themes were identified through interviews with staff: *A need for holiday provision; Social outlet; Help beyond breakfast clubs; Addressing need; Staffing and expertise; and Planning and advertising*. Each theme is subsequently discussed in detail with supporting quotes from staff.

#### Theme 1: A Need for Holiday Breakfast Provision

Staff proposed that many families face financial difficulties, which results in parents struggling to provide a sufficient supply of food for their children:

They haven’t got the money to buy the food, they’ve gotta pay out for essentials like rent, housing tax if they have to pay housing tax, electric, gas, all the bills that need paying and of course by the time they’ve paid all them they haven’t got the finances to support children to have breakfast or dinner (Female staff member; Club 3)

The summer break from school was suggested to add further pressure to families as they need to provide additional meals for their children that they would not usually provide during term time. The summer break was thought to be considerably challenging for those entitled to free school meals during term time:

Some of the kids are on free school dinners as well and part of the reason – not like the main reason, but part of the reason for doing it was six weeks without a breakfast or dinner that you’ve previously been having for free urm was part of the motivation for doing it (Female staff member; Club 2)

Holiday breakfast clubs were thought to support children and families by providing a reliable source of breakfast throughout the summer break:

Making sure children have had breakfast definitely making sure they’ve had something to eat ‘cause some of them probably wouldn’t have had (Female staff member; Club 1)

Access to a breakfast meal was believed to be beneficial to the breakfast intake of those who attended holiday breakfast clubs. The breakfast meal provided a more varied and substantial breakfast meal than people would have access to at home:

I think at least it would give the kids a chance to have a decent meal to start the day off with ‘cause they might not have anything – no I’m not saying they not gonna have anything else but they may not have anything substantial again throughout the day you know so I think it makes a good start to the day for them (Male staff member; Club 4)

Additionally, it was proposed that attendees would be more likely to eat breakfast at breakfast club and would consume healthier items than they would have at home:

At home you know they’ll have a slice of toast that they won’t even finish or they’ll wander around and never eat whereas when they’re mixing with other kids they tend to sit and do and eat and not think about what they’re eating (Female staff member; Club 3)

They’ve started to eat fruit for us but they didn’t always eat fruit you know (Female staff member; Club 6)

One member of staff also pointed out that holiday breakfast clubs allow everyone to access a breakfast meal, i.e., adults as well as children, which was viewed as important:

Main thing is for the kids but I think it’s really benefitted the adults as well so urm yeah just making sure everyone’s getting some food, which is really important ’cause breakfast, the most important meal of the day (Female staff member; Club 5)

Staff recognized the value of the holiday breakfast schemes within their areas and felt that the scheme should be implemented on a wider scale in future:

It would be great if we could actually do this urm every kind of holiday you know that they have a period off school not only a day or two but if they had say a week or two weeks off school it’d be great to do this (Female staff member; Club 1)

It was anticipated that greater availability of holiday breakfast provision would result in greater numbers of attendees:

There is a lot of poverty in [town] I think they’ve only touched the surface of it here urm it has came on pretty quick you know to get it set up we’d have probably maybe a hundred wee’uns in here if, you know, if we had the facility to let them in ‘cause in the food bank alone we have over a thousand people come through (Female staff member; Club 6)

Even in those clubs that had been less successful at attracting attendees, staff felt that it was important to address the issue of non-attendance and offer the holiday breakfast provision again in future:

I’m not put – you know – not put off, we tried you know it’s not all throw it in it didn’t work because there are always ways (Female staff member; Club 4)

However, future implementation of holiday breakfast clubs would require careful planning as staff advised that there is an element of suspicion surrounding the provision of free breakfast and a perception that free breakfast clubs are for poor people. This was the case even when the clubs had been advertised universally and were not targeted at those with particular needs:

I got the feeling that – some of the comments made today – I mentioned it to older people that we have the breakfast club – is er is it just for people who are really poor or is it just for people who’ve got nothing and urm I don’t think it was put across like that, it wasn’t necessarily – that’s not what it was about really (Female staff member; Club 4)

Some people are quite suspicious when it’s free, they go how much does it cost, you go no it’s free, why is it free? Do you know what I mean it’s quite sort of funny because if we’d have charged fifty p then we’d have ­probably had more people come (Female staff member; Club 3)

One staff member felt quite strongly about maintaining the universal element of the breakfast clubs. She believed that providing a breakfast club for all families regardless of income was a successful way of reducing stigma and the introduction of inclusion criteria could reduce the numbers of people attending:

I think if you start to say to people you can only come if you earn less than sixteen grand then – then or whatever you know whatever arbitrary amount it is – why would you, you know that’s then almost … that’s – that’s – it’s not something I’m comfortable with because you don’t know the size of anyone’s mortgage car loan credit card debt you don’t know how – how much someone’s struggling socially regardless of how things look on the outside … I think if you did start to put rules on it and say you can only come if you earn more than, if you earn less then you won’t get anyone will you in my view (Female staff member; Club 2)

Furthermore, it was suggested that providing food to families in need through a free community breakfast club was less stigmatized than other methods of providing food aid, particularly for children:

This is helping to take away the stigma of the food bank because it does take a lot for our parents to come to the food bank but it’s been phenomenal for what the stigma – sleepless nights I’ve had trying to think of ways to do this and here it is now (Female staff member; Club 6)

#### Theme 2: Social Outlet

Staff were keen to stress that holiday breakfast clubs were not only used as a means of obtaining a breakfast meal but also used by some as a place to meet others too:

I think a lot of it’s for people to come in and have a chat as well urm as well as having breakfast (Female staff member; Club 5)

This was the case for adults as well as children:

They interact with other children and of course parents interact with other parents (Female staff member; Club 3)

Breakfast clubs were thought to be particularly beneficial for children as they allowed them to meet up with school friends they might otherwise be unable to spend time with throughout the summer break:

Keeping the children urm they get together again you know through school you miss your mates at school don’t you when you’re off for the six weeks so there’s all that social side involved in this as well (Female staff member; Club 4)

Children in one club, where activities were offered within the community, used breakfast club as a central meeting place. They would meet with their friends at breakfast club, have breakfast then go onto do community activities:

Quite a few of them are using it as a meeting place as well ‘cause they go off and doing other activities so quite a lot of them meet here, have their breakfast and then they go off and do like swimming and horse riding and all sorts like that (Female staff member; Club 1)

The social time that children were able to spend together was thought by staff to have benefited children’s social skills during the school holidays:

It’s yes and thank you and they wait their turn now to speak (Female staff member; Club 6)

Staff also recognized holiday breakfast provision as a useful way of bringing families and community members together:

I think that everybody just thinks finally the estate’s come to life during the six weeks holidays rather than everybody staying inside with their doors shut it really has been like part of the community (Female staff member; Club 1)

Bringing people together in a community space was thought to reduce isolation that people often encounter, particularly during the school holidays:

When you talk to people they just want some company, somewhere to go, a purpose (Female staff member; Club 5)

The lady said if the breakfast club wasn’t here she said she’d actually be stuck in her house all the time (Female staff member; Club 1)

Isolation was thought to be an issue that numerous families faced during the school holidays as many of the activities available for families incur a cost thus parents are restricted in what they are able to do with their children:

There is isolation, isolation for the kids in the summertime urm not being part of the school urm parents and guardians not having enough money because some of the clubs, if not all of the clubs, which I have looked into, some of the summer schemes you have to pay them (Female staff member; Club 6)

The provision of free breakfast clubs gave families an accessible activity to attend each day:

I think it’s a bit of a no brainer isn’t it, regardless of your financial situation it’s a long holiday and it’s nice just to have something to go to (Female staff member; Club 2)

#### Theme 3: Help Beyond Breakfast Clubs

Staff suggested that it can be quite difficult for people to access community organizations and to obtain necessary help through external agencies. Holiday breakfast clubs were thought to support families to become involved in community groups and to access the help they need beyond breakfast provision:

Sometimes going to a community center is quite daunting isn’t it … I think it breaks down those barriers because they go to a child’s activity but they’ve stepped across the door then and they’ve met the staff and they’ve met the volunteers (Female staff member; Club 3)

Breakfast clubs were able to work closely with other external agencies to sign post people in need of food provision to the holiday breakfast clubs:

Spoke to the home school community liaison people to say this is on so anyone who you know who’s reliant on a free school meal … and with the food bank as well we targeted the food bank yeah so the food bank have been referring people on (Female staff member; Club 2)

Staff felt that once people accessed holiday breakfast clubs, they could be offered additional support within the organization providing the breakfast club:

They’re doing like a clothes bank as well they’ve – urm which is great which whoever comes here will hear about as well so opportunity for them to get clothes things like that as well and then they can hear about the foodbank so if they’re struggling (Female staff member; Club 5)

Support could also be offered to help people to access other groups and external organizations who could address specific needs:

Some of our parents are – have already signed up to our unemployment classes for September for doing computer courses and stuff like that so it really does open it up (Female staff member; Club 5)

It was further suggested that holiday breakfast clubs could potentially act as a starting point for the development of parental support groups, which could bring parents together in similar situations to offer support to one another within a social environment outside the breakfast club:

Get a little group up themselves, start you know having groups and discussing what they could do like help each other and things like that (Female staff member; Club 3)

#### Theme 4: Addressing Need

Discussions with staff revealed that they had a unique insight into the needs of people in their local area and they believed that addressing the needs of the people in their areas would result in a greater number of people attending holiday breakfast clubs:

Get some feedback on what would be appropriate and they could embrace – engage with – it may be that they – we don’t know until you ask them … maybe the next time we might need to ask the pare – ask the community (Female staff member; Club 4)

It was evident that it would not be practical to implement a single breakfast club model across all areas without considering the needs of the people living in those areas:

Different parts of the country, different towns, different parts of [city] you will have a different result from what you have – and it’s the social area, the deprivation will make a difference (Male staff member; Club 4)

For example, some of the breakfast clubs required children to be accompanied by an adult and these clubs had been well attended. However, some staff believed that a requirement for children to attend breakfast clubs with an adult might have hindered some children from accessing the provision:

There’s a lot of parents that don’t want to you know take part in different things with their kids (Female staff member; Club 1)

Moreover, it was suggested that some children would have been reluctant to attend breakfast club with their parents, which again could have acted as a barrier to attendance:

I think there’s a lot of children that wouldn’t have come if they had to bring an adult with them definitely (Female staff member; Club 1)

In relation to addressing the needs of the people within local areas, staff believed that the environment in which the breakfast clubs took place made a substantial difference to the way that the breakfast club was perceived and the activities that could be offered. For some clubs, a lack of space had limited what they had been able to offer:

It might be nice if we had a bigger building urm ‘cause originally we were going to use the school but there’s loads of building work being done so we couldn’t use school but possibly if we do it again in the next holidays we could possibly use the school hall because we’ve got limited spaces we’re having to send people away like when they’ve finished eating (Female staff member; Club 1)

Where space was less restrictive, a wider variety of activities could be provided:

I think the activities made a massive difference having something that people like we’ll go and have a kick about and it almost is an accessible – there’s something gone on in the park every day that has made a big difference so if we were doing it again we would do that (Female staff member; Club 2)

Moreover, some clubs encountered difficulty with the utilization of buildings that were predominately used for other purposes:

The unfortunate thing this is not our premises we can’t do what we like in it we could do a lot lot more urm we had to cut off last week because the hall was pre-booked and the kids did miss it (Female staff member; Club 6)

Additionally, it was suggested that people can have pre-conceived ideas about particular venues, which subsequently determines their views about the types of activities that take place within such venues:

People are a bit reluctant to come into a church … our hall is connected to the church and cause it’s connected to the church they think it’s church orientated (Female staff member; Club 3)

It is therefore important that the breakfast club venue is carefully considered:

One of the things that we also felt that wouldn’t work would be the dignity for the children because we were actually in the food bank premises so we brought it then to our local parish hall which we’re very lucky to get the upstairs space it has worked fantastically (Female staff member; Club 6)

#### Theme 5: Staffing and Expertise

All holiday breakfast clubs were supervised by at least two members of staff at all times and in some cases, as well as attending with their children, parents helped to support breakfast club staff by clearing tables and supervising activities:

We’ve had enough volunteers like me and [breakfast club staff] have been here most days erm doing it between us but then other people will pop in and do a load of washing up or will come in and do the toast and stuff like that (Female staff member; Club 2)

However, it was suggested that staff training and the sharing of best practice among clubs in different areas would be beneficial to the development of holiday breakfast clubs in the future:

We would look to develop ourselves as in training courses and that’s for the better of the kids (Female staff member; Club 6)

There’s probably quite a lot of expertise around on running holiday activities including breakfast clubs so maybe that needs to be an issue for this project as well, how information is disseminated … I think to share the information otherwise we don’t know what’s working or what they found was most helpful (Female staff member; Club 4)

Some clubs had successfully drawn on the expertise of volunteers within their local communities to offer activities to children as part of the breakfast club. This worked well for those who had utilized such provision and was recognized as a potential area for further development:

Three days a week we had or we have people coming in to do junk modelling and craft sessions like crocheting and junk modelling and stuff like that and they’re volunteers who’ve realised what we’re doing and said I’m a primary school teacher I can come in once a week and then nature rangers as well which is another local parent where she’s come in and done like tree identification (Female staff member; Club 2)

We probably could do more with interacting with people outside as in taking them on field trips and stuff like that, that would be something you know that you would look to do because there’s so many people out there looking to help and say bring the kids along (Female staff member; Club 6)

#### Theme 6: Planning and Advertising

A final area outlined by staff, which is a key consideration for the future development of holiday breakfast clubs, is timely and appropriate advertising. Staff felt that the breakfast clubs had been implemented quite quickly, which left them very little time to let people know about the breakfast club:

If we’d probably known a bit sooner before the end of the school holidays we might have been able to you know promote it you know quite a few weeks before … yeah just a little bit more planning I suppose at the beginning (Male staff member; Club 4)

Though limited planning time had not hindered attendance at all clubs:

The first couple of days was hard cause all of a sudden we had a hundred people turn up at half past nine in the morning I was like aaaaah! What! But erm you know since we’ve got used to that that’s been ok (Female staff member; Club 2)

Staff believed that more time to promote the clubs would have resulted in greater numbers of families being reached:

Roll it out through the schools more and that will probably bring bigger numbers (Female staff member; Club 6)

### What are the uses, impacts and areas for future development of holiday breakfast clubs according to adult attendees?

The adult participant group identified a number of uses, impacts and potential areas for the development of holiday breakfast clubs. The views of adult participants are crucial in the evaluation of community interventions as they can comment on the direct impact of such interventions on their home lives and can verify whether staff ideas about the needs of families in their local communities are accurate.

Five key themes were evident through discussions with adult attendees: *A need for holiday breakfast provision; Social outlet; Financial implications; Provision of routine; and Planning and advertising*. Themes are subsequently discussed with quotes from adult attendees presented to illustrate each theme.

#### Theme 1: A Need for Holiday Breakfast Provision

Supporting the views of breakfast club staff, it was clear from discussions with parents that some struggled to feed their families. However, they were very keen to stress that they endeavored to provide their children with an adequate supply of food and adopted different strategies to ensure their children could eat including meal skipping themselves and sharing food with family and friends:

Some days I couldn’t eat at all if it was just enough for [child] he’d have it, I’m like that, I’d rather starve than not let [child] have anything. [Child] will always come first, always (Parent; Club 5)

I feed my son and I go without a lot of the time or I share with my neighbor one night she’ll cook the next night I cook to help us both out food wise (Parent; Club 1)

Parents placed high importance on ensuring their children had what they considered to be an appropriate quantity of food and this took precedence over the provision of a nutritionally balanced meal:

I’ve always made sure my children have meals, whether or not they’re balanced is another matter I mean you – even if it’s just noodles and hotdogs you know not very balanced but it’s a nice filling meal isn’t it (Parent; Club 3)

Adult attendees participated in breakfast clubs to have a breakfast meal themselves or to allow their children to have breakfast:

To have something to eat firstly and to join in (Parent; Club 1)

For some, attendance was self-motivated while others had been referred to the breakfast club by external agents, such as carers, schools, or social services, to access the breakfast that was freely available:

I got the token off the school (Parent; Club 5)

It was my social worker who referred me and thought it would be a good idea for the children (Parent; Club 6)

The varied breakfast menu available to all breakfast club attendees was highly valued among participants and something that was highlighted even by those who did not attend specifically to access a breakfast meal:

At home we tend to buy the same cereal urm they tend to every morning that’s the particular cereal they want but with the breakfast club what I’ve noticed is because there’s urm a few more on offer they will actually vary what they have (Parent; Club 3)

The breakfast served at breakfast club allowed participants to consume a range of breakfast foods, which was much more varied and in some cases healthier than the breakfast consumed at home:

I’d be a bit embarrassed saying it. I wouldn’t have a lot of money so what they would have here it’s stuff what I’ve always wanted to buy and they’ve asked for and I can’t afford it so I say no you can’t have that we’ll get something else so it’s good that way they’re getting to try different things (Parent; Club 6)

With everything else it’s cut out all the frying stuff and that which is good (Single adult; Club 5)

For one parent, the breakfast club also offered some peace of mind as the varied breakfast menu was guaranteed to be available:

The day before pay day can be tough as you know and it’s – they don’t run out, they don’t run out of cereal or they don’t run out of milk or they don’t run out of bread and so they’ve got the choice there all the time whereas they wouldn’t necessarily at home (Parent; Club 3)

Additionally, the breakfast club was suggested to encourage a positive breakfast habit among both adults and children, some of whom would have skipped breakfast had the breakfast club not been available:

No, no when I’m at home I wouldn’t bother ‘cause I’m not a breakfast person (Parent; Club 5)

They don’t miss it, it doesn’t matter what time they got to bed at … they set their own clock as like to time to make sure they’re up give them time to get dressed and actually get here for breakfast club (Parent; Club 1)

Like staff, adult attendees recognized the value of holiday breakfast provision and hoped that the scheme would be continued in future:

I hope you do it again because it is a good thing (Single adult; Club 5)

The only thing that I would like is if it was continued say every mid-term stuff like that if it was a regular – not just summer but every holiday they have from school because not only with mine but with a lot of ‘em you see kids on the street at quarter past eight in the morning and if they didn’t have here to come what are they doing (Parent; Club 1)

If the scheme was to be implemented again, it was suggested that it should be available for a longer duration each day:

There are a lot of other people don’t get up earlier so perhaps making it sort of a two hour time frame around nine till eleven urm but then have it more of a kind of drop in feel to it ‘cause I think for some I think feels like oh we’ve got to be there at quarter past nine no later no earlier urm and then you’ve gotta be out and finished by quarter past ten so it’s like you know some people think oh you know I’ve gotta rush to get going or you know I’ve gotta rush to get there (Parent; Club 3)

#### Theme 2: Social Outlet

All adults emphasized the social value of breakfast club attendance. Some were keen to stress that for them, breakfast was a secondary reason for attendance at breakfast club and they mainly attended for the social opportunities available:

It’s a chance for the children to catch up with their friends because they’ve got a lot of friends that come as well and it’s a chance for me to catch up with other parents urm I think the breakfast part of it is second to that (Parent; Club 3)

Adults had been able to spend time with members of their local area that they would not typically have interacted with:

It’s done a really lot of good to me ‘cause coming out and meeting other people and then meeting other friends you see but not get to talk to ‘cause they’re always in a rush going to school with the ki – the children and it’s great when you’re all together in here ‘cause you can sit down you can talk you can stay quite a lo – you can stay for a while you don’t have to get out and rush (Single adult; Club 5)

Time spent together had allowed new friendships to form:

I’ve been able to get to know some of the other mum’s a bit more especially some that are going – whose children are going into school with [child] so it’s been nice and numbers have been exchanged and things (Parent; Club 2)

Myself, I’ve made some new friends through it, I think you need friends at the end of the day and I don’t think I’ve ever had that but now (Parent; Club 5)

Similarly, adults emphasized the value of the breakfast club in allowing children to make new friends as well as spending time with school friends that they would not usually see during the school holidays:

[child] meets her friends where she wouldn’t see them normally at the school holidays (Parent; Club 1)

I started coming with a particular child because she’s just finished nursery going into the next stage of school and it was agreed with her friends from the nursery to meet at the breakfast club to keep the friendships going ready for school (Child minder; Club 2)

Furthermore, holiday breakfast clubs reduced isolation for children and adults during the summer break. A lack of suitable activities and a lack of routine gave families the potential to become quite isolated during the long summer break:

I think I would have struggled to find activities to amuse him probably because it would have been just me and him whereas here he gets the chance to just play with other kids and he’s quite a sociable child so he’s quite happy (Parent; Club 2)

I suffer a wee bit from depression so I think it’s good for them to be getting out and about and for myself to be getting out of the house as well you know (Parent; Club 6)

Similarly, adult participants without children also suggested that breakfast club had reduced their tendency to remain at home alone:

I’d stay in because I couldn’t do the time that I’m here around the shops or around anywhere I can’t do this length of time doing anything apart from sitting or being a bit more comfortable you know so it’s ideal for me personally (Single adult; Club 5)

I live on my own, saves me cooking ‘cause you get fed up with your own cooking and that and you get fed up with your own – on your own and that so it’s a great idea to come up and meet friends (Single adult; Club 5)

Further to reducing isolation, breakfast clubs were thought to act as community hub bringing people together in one place:

This place is kind of central and we kind of amalgamate here to see each other (Parent; Club 3)

There could be other stuff going on in the area that the kids wouldn’t know about then one of the other kids saying are you going such and such a place today so it’s a place for information as well (Parent; Club 1)

#### Theme 3: Financial Implications

It was evident that some parents faced financial difficulties, particularly during the school holidays, which limited their capacity to be able to confidently provide their families with an adequate and varied supply of food:

We do be a bit under pressure you know financially with them being off school (Parent; Club 6)

Obviously it’s hard because you have to buy more food in for the holidays because when they’re at school they get school meals so it’s harder to know what you need in the course of a week so therefore it ends up costing more (Parent; Club 3)

However, the provision of breakfast helped parents to save money and made food in the home last longer than it would usually:

I work for an agency where I don’t get paid in the school holidays the money’s a bit more – it’s tight ‘cause it’s school holidays so having your breakfast free here releases that little bit of extra money at the end of the week (Parent; Club 1)

It saves on milk as well and milk’s not incredibly cheap nowadays … probably save about two boxes of cereal a week by them coming here so that is a brilliant thing (Parent; Club 1)

For some who were attending the breakfast club but did not feel that they needed to access the breakfast meal provided, they expressed a desire to find a way to give back to the organization running the breakfast club through means of a financial donation or by spending money in a way that would benefit the organization:

I know that I’ve saved on the breakfast but what I’ve saved on the breakfast I’ll spend in the church and the shop … so I don’t feel like I’ve had something for nothing if you know what I mean I feel like I haven’t really paid my way but I haven’t not paid my way either because I’ve give something back (Single adult; Club 5)

#### Theme 4: Provision of Routine

There was a consensus among parents that the school holidays led to a lack of routine within their families. Parents talked about the way in which children tended to sleep in later during the holidays, which led to a more sporadic eating pattern and a habit of skipping breakfast because they were waking up closer to lunchtime:

During the school holidays because they go to sleep later they get up later so by the time they’re getting up it’s like lunchtime rather than breakfast time so my eldest doesn’t eat breakfast at all, I don’t really – I won’t eat breakfast during the summer and then they’ve got a really bad eating habit actually during the holidays yeah so it’s like mealtimes are never regular and it’s more so they don’t have breakfast (Parent; Club 1)

The lack of routine during the holidays was a concern for some parents as they had experienced difficulties in the past with getting children back into a routine of getting up early when they returned to school:

Routine, because they do lack it during the summer then it’s hard when they start back in school, it’s really hard to try and get them back into a routine” (Parent; Club 1)

The provision of a breakfast club was thought to help families to maintain a consistent routine between holidays and term time. Moreover, parents believed that this would make things easier for their children to fall back into a school routine following the holidays:

They do a breakfast club at school so it’s kinda followed on from that which is a good thing, gets them up so they’re not lounging in bed (Parent; Club 1)

It just gives them – knowing that they’ll be in a routine for getting up for their breakfast before school and eating a proper breakfast before school you know (Parent; Club 6)

#### Theme 5: Planning and Advertising

Like staff, adult attendees felt that future holiday breakfast clubs could benefit from more advertising to attract greater numbers of attendees:

In all honesty I think for this area it came about a bit too late because it was a bit of a rush to sort of get it hosted erm for future years I think obviously we’ll obviously be able to plan a bit more and advertise it a bit more openly (Parent; Club 3)

People round here who I know I think they’re a bit scared of putting themselves forward for anything around here, it needs to be more enticing for them (Parent; Club 1)

### What are the uses, impacts and areas for future development of holiday breakfast clubs according to children?

It has been argued that the views of children are just as crucial as those of adults and should be considered in the development of breakfast club provision (25).

Three key themes were identified through discussions with children: *A need for holiday breakfast provision; Social outlet; and More activities*. Themes are subsequently discussed with quotes from children presented to support each theme.

#### Theme 1: A Need for Holiday Breakfast Provision

As suggested by adult attendees and breakfast club staff, holiday breakfast clubs were used by children to obtain a breakfast meal. There were no suggestions made by any children that breakfast was unavailable at home but all children who participated in the current study mentioned that they had breakfast at breakfast club:

Usually I’ll have toast or cereal for breakfast (Male; Club 2)

Attendance at holiday breakfast clubs provided children with access to a wider variety of breakfast foods than they would be able to have at home:

Breakfast bars and a wee glass of diluted orange or apple juice or blackcurrant and fruit (Male; Club 6)

This variety allowed children to try new foods that they had not tried before:

Interviewer: *Have you had any foods or drinks at breakfast club that you’d never tasted before?*

Child: *Apple juice* (Female; Club 1)

Additionally, one child mentioned that she was more likely to consume breakfast at breakfast club because she enjoyed the food more at breakfast club than at home:

Interviewer: *Why do you have toast here and not at home?*

Child: *‘Cause they make it better* (Female; Club 1)

Moreover, another child suggested that he had more of an opportunity to sit down to breakfast at breakfast club without feeling rushed as he did at home:

*At home, we like rush around a bit and we don’t really eat it* (Male; Club 1)

All children enjoyed attending breakfast club and all but one child said that they would like to be able to attend holiday breakfast clubs in future:

Interviewer: *And why would you like to come along?*

Child: *Because it’s really fun* (Male; Club 3)

The individual child who said she would not like to attend again in future suggested that she would not like to attend because she felt too rushed in the morning to get to breakfast club:

Because my dad rushes me (Female; Club 1)

However, children were overall very positive about holiday breakfast clubs and one child hoped that more children could attend:

Like attracts more people and stuff (Female; Club 1)

#### Theme 2: Social Outlet

For most children, the provision of activities and the opportunities to meet with friends also attracted them to attend breakfast club:

I like painting and making loom bands (Female; Club 6)

The time that children were able to spend interacting with peers in breakfast clubs was highly valued. Children talked about how participation in breakfast club allowed them to make new friends:

Well I didn’t have friends the first time it started and then when people started coming I joined friends with them (Male; Club 2)

Also, children were able to spend time with existing friends that they would not usually be able to spend time with during the long summer break:

Some of my friends who live like up in [area] yeah they come down here to have breakfast club (Male; Club 1)

As mentioned by adult attendees and staff, holiday breakfast clubs helped to reduce isolation during the summer break:

I’ve got one friend who usually just sits at home playing on his XBox but he’s been coming (Male; Club 1)

My mum thinks it’s brilliant, love what they’re doing cause last six weeks holiday I didn’t really do anything (Female; Club 1)

In addition, breakfast clubs were suggested to bring families together:

Play with my little cousin a lot more (Female Club 1)

#### Theme 3: More Activities

Finally, children’s suggestions for improvements to holiday breakfast clubs centered around the inclusion of more activities. Children did not express any concerns about the way the breakfast clubs were running at the time nor did they suggest that there were issues with a lack of resources; they simply requested more when asked what could be improved:

Have like – have the computers at the back up and running so you can turn it into like an internet café (Male; Club 1)

I know! A party every day the breakfast club’s on (Female; Club 6)

## Discussion

The current evaluation set out to investigate the uses, impacts, and potential areas for development of holiday breakfast clubs in the UK. Findings highlighted a need for holiday breakfast provision and revealed a multitude of benefits of holiday breakfast clubs as well as potential areas for development and broader implementation of holiday breakfast schemes. First, the evaluation demonstrated that food insecurity is an issue for many families, which is exacerbated during the long summer break when parents are unable to rely on school meals as a consistent source of food for their children. Having to provide additional food for their families during the summer holidays often placed parents under financial strain but it was evident that parents felt strongly about doing everything they could to ensure their children ate what they considered to be an adequate amount of food. Parents adopted a range of strategies to ensure their children ate enough including reducing their own food intake by cutting portions or skipping meals completely, compromising on the nutritional balance of meals by providing a greater quantity of cheaper, less nutritious foods and accepting support from family, friends, and external agencies, such as food banks.

It was recently acknowledged in a report by the All Party Parliamentary Group (APPG) on Hunger and Food Poverty (26) that hunger is a widespread issue within the UK. It was argued within the report that there are “too many people” (p. 8) struggling to afford to be able to adequately feed themselves and many of those who can afford food are living on a limited diet consisting of a small range of foods. The findings of the current investigation lend support to the APPG (26) report by providing further evidence of food insecurity and illustrating the strategies used by families to counteract hunger, some of which have the potential to be detrimental to the health of both children and parents. Moreover, these findings mirror those of a previous investigation carried out by Gill and Sharma (13) who reported that the school summer break can put families under considerable financial difficulty leading to a reliance on a small range of cheap, less nutritious foods. The current investigation highlights the need to address the issue of food insecurity during the school holidays as it is evident that families are still facing the same difficulties more than 10 years after the issue was highlighted by Gill and Sharma (13).

The results of the current study showed that holiday breakfast clubs could support families during the school holidays by offering consistent access to a broad range of nutritious breakfast foods. The varied menu offered within the holiday breakfast clubs was valued by children and adults as it allowed them to consume a more diverse range of foods than they would have access to at home. Previous research has shown that access to breakfast within a breakfast club setting can result in children being more willing to try new foods (19) and lead them to consume a higher proportion of healthy breakfast items than children who do not attend breakfast club (10, 11). In a similar vein, the varied breakfast menu provided within holiday breakfast clubs could contribute positively to the nutritional needs of children and adults by ensuring that they have access to an array of breakfast foods including cereals, fruits, bread items, dairy products, and fruit juices; access to which is more limited at home.

Furthermore, in relation to the impact of holiday breakfast provision on family food circumstances, some adults suggested that access to breakfast at holiday breakfast clubs meant that they had to spend less on breakfast at home and meant that some food items lasted longer than they would usually. Given that food and drink costs have risen by 47% over the last 10 years (27) while increases in wages and benefits have occurred at a much slower rate (28), the provision of a breakfast meal for families has the potential to make a considerable difference to family food and finances. Moreover, 66% of children living in poverty have at least one working parent (29) thus offering holiday breakfast schemes universally, i.e., with no pre-determined attendance criteria maybe beneficial in some areas where poverty is evident among working communities.

It has been argued that the process of accessing food aid within the UK is problematic as it involves a process of referral, which highlights the needs and vulnerabilities of those looking to access free food provision and consequently provides an experience that is contradictory to the process of choosing and purchasing food that is typical within UK society (30). The provision of universal free breakfast clubs during school holidays might support those individuals most in need to access food provision in a manner that is perceived to be more socially acceptable than the food aid referral route. Holiday breakfast clubs that are universally free support a right to food approach, which specifies that the need of anyone who cannot provide food for themselves should be fulfilled (30). By providing food with no access criteria, holiday breakfast clubs ensure that food can be accessed by all regardless of circumstance.

In addition to the potential impact of the food provision, holiday breakfast club attendance was associated with a variety of additional social and behavioral benefits for children and adults. It was evident that families typically lacked routine during the summer break, which resulted in missed meals and the potential to become isolated and sedentary. Holiday breakfast clubs helped to counteract this potential for isolation and helped children to maintain a routine of getting up in the morning, which parents believed would make the school routine easier when children returned after the holidays. Moreover, the social element of holiday breakfast clubs was highly valued. Attendance at breakfast clubs allowed adults and children to build new friendships as well as providing children with opportunities to meet with school friends that they would not typically be able to spend time with during the summer holidays. The recent report by the APPG (26) suggested that many of the social outlets that people used to rely on (i.e., extended family, neighbors) no longer exist resulting in a lack of adequate support in times of need. If holiday breakfast clubs offer an environment that encourages the development of positive relationships then this could result in greater support networks within local communities. Holiday breakfast clubs were thought to bring communities together and offer a place where people can receive additional support and information beyond breakfast provision thus highlighting the potential wider reaching benefits of holiday breakfast clubs that could be maintained beyond the life of the clubs. Furthermore, the utilization of skills of local community members (e.g., sports leadership, crafts) was recognized as a useful way of offering a variety of activities to children and families through the breakfast clubs thus demonstrating further potential for drawing on community support that could continue beyond the summer months.

While the breakfast clubs were viewed positively by children, staff, and adult participants, there were suggestions made for potential areas of development and improvement. First, the breakfast club setting had a considerable impact on the services that were offered through the breakfast club and may also have influenced the levels of attendance at each club. The most popular clubs, according to number of attendees, appeared to be those based in neutral community venues (i.e., community buildings; local parks). While the church-based club had attracted families to attend, a member of staff speculated that some people may have been put off attending due to pre-conceived ideas that a church-based club is linked to religious activity, even though this was not the case. Similarly, clubs based within food banks had struggled to attract attendees, which is unfortunate given that a member of breakfast club staff suggested that holiday breakfast clubs could offer a useful outlet for providing food to families in need while reducing the stigma that is often associated with direct use of food banks. It was suggested that a food bank venue could lead people to associate the breakfast club with being poor and having a specific need for free food thus highlighting the importance of clear communication between breakfast clubs and potential attendees to ensure that the club’s aims and activities are understood allowing people to make a more informed decision on whether to participate. Previous research has shown that a breakfast club model can be implemented across different venues but the needs of individual areas should be considered so that the breakfast club model can be adapted to suit local needs accordingly (19). It would be useful in future evaluations of holiday food provision to investigate the views of people within local communities who actively choose not to attend holiday clubs. This would provide an insight into the barriers to accessing holiday food provision and highlight important areas for consideration by those involved in the set-up of holiday food schemes.

Overall, the findings of the current investigation provide a useful and timely contribution to the research literature by offering the first academic evaluation of holiday breakfast provision in the UK. The study demonstrates a triangulation of views by presenting evidence from children, adult attendees, and breakfast club staff to ensure that the opinions of key users and stakeholders directly involved in holiday breakfast clubs on a daily basis are represented.

It is important to acknowledge that the current study utilized a relatively small sample of participants based predominantly in the North West of England, thus the findings might not generalize to other areas of the UK. However, the findings provide a useful starting point for further investigation and development of holiday food provision. Given that some of the impacts of holiday breakfast clubs outlined within the present study mirror those of previous investigations that have found breakfast clubs to be particularly advantageous to nutritional and social outcomes (10, 11, 19, 20, 31), future research should begin to quantitatively measure the impact of both term time and holiday breakfast clubs to consider whether attendance is associated with changes in health, social, financial, and educational outcomes.

To conclude, the findings of the current investigation provide evidence of food insecurity in the UK and suggest that holiday breakfast clubs could reduce the burden placed on families during school holidays. Moreover, holiday breakfast clubs confer a number of benefits that could impact on the health and wellbeing of children and adults and make the transition between term time and holiday time much less pressured for families. The views of participants in the current study should be drawn upon to inform future development and implementation of holiday food provision in the UK and to investigate the potentially wider benefits of such provision for families and communities.

## Conflict of Interest Statement

The research was funded by Kellogg’s.

## Footnotes

^1^http://www.un.org/Overview/rights.html

^2^http://bestpractices.nokidhungry.org/summer-meals/about-summer-meals
